# Gold nanoparticles as high-resolution X-ray imaging contrast agents for the analysis of tumor-related micro-vasculature

**DOI:** 10.1186/1477-3155-10-10

**Published:** 2012-03-12

**Authors:** Chia-Chi Chien, Hsiang-Hsin Chen, Sheng-Feng Lai, Kang-Chao Wu, Xiaoqing Cai, Yeukuang Hwu, Cyril Petibois, Yong Chu, Giorgio Margaritondo

**Affiliations:** 1 Institute of Physics, Academia Sinica, Nankang, Taipei 115, Taiwan; 2 Department of Engineering and System Science, National Tsing Hua University, Hsinchu 300, Taiwan; 3 Department of Otolaryngology-Head and Neck surgery, Mackay Memorial Hospital Hsinchu Branch, Hsinchu 300, Taiwan; 4 Advanced Optoelectronic Technology Center, National Cheng Kung University, Tainan 701, Taiwan; 5 Université de Bordeaux, CNRS UMR 5248 - CBMN, F33405 Talence-Cedex, France; 6 National Synchrotron Light Source-II, Brookhaven National Laboratory, Upton, NY, USA; 7 Ecole Polytechnique Fédérale de Lausanne (EPFL), CH-1015 Lausanne, Switzerland

**Keywords:** Synchrotron, X-rays, Angiography, Angiogenesis, Contrast, Au Nanoparticles, Heparin

## Abstract

**Background:**

Angiogenesis is widely investigated in conjunction with cancer development, in particular because of the possibility of early stage detection and of new therapeutic strategies. However, such studies are negatively affected by the limitations of imaging techniques in the detection of microscopic blood vessels (diameter 3-5 μm) grown under angiogenic stress. We report that synchrotron-based X-ray imaging techniques with very high spatial resolution can overcome this obstacle, provided that suitable contrast agents are used.

**Results:**

We tested different contrast agents based on gold nanoparticles (AuNPs) for the detection of cancer-related angiogenesis by synchrotron microradiology, microtomography and high resolution X-ray microscopy. Among them only bare-AuNPs in conjunction with heparin injection provided sufficient contrast to allow *in vivo *detection of small capillary species (the smallest measured lumen diameters were 3-5 μm). The detected vessel density was 3-7 times higher than with other nanoparticles. We also found that bare-AuNPs with heparin allows detecting symptoms of local extravascular nanoparticle diffusion in tumor areas where capillary leakage appeared.

**Conclusions:**

Although high-Z AuNPs are natural candidates as radiology contrast agents, their success is not guaranteed, in particular when targeting very small blood vessels in tumor-related angiography. We found that AuNPs injected with heparin produced the contrast level needed to reveal--for the first time by X-ray imaging--tumor microvessels with 3-5 μm diameter as well as extravascular diffusion due to basal membrane defenestration. These results open the interesting possibility of functional imaging of the tumor microvasculature, of its development and organization, as well as of the effects of anti-angiogenic drugs.

## Background

Limited contrast has been a crucial problem in radiology since the discovery of X-rays [1]. The problem is particularly acute in the imaging of small blood vessels [2], in particular in the detection of vascular angiogenesis, critical for the early diagnosis of cancer [3], notably for tumors becoming malignant after vascularization.

This is a relevant issue: angiogenesis is widely investigated in conjunction with cancer development [4-8], and could lead to early detection and new therapeutic strategies [9,10]. However, such studies are negatively impacted by the limitations of established imaging techniques in the detection of micro-vessels. New approaches, synchrotron-based X-ray micro-radiology and micro-tomography, were recently tested for angiography studies [2,3,11-13]. Effective contrast agents are highly desirable for these techniques.

Finding such agents is therefore a prime objective, in particular for nanotechnology. Nanoparticles and other nanosystems are indeed increasingly investigated as contrast agents for radiology. However, the effectiveness of different types of nanoparticles in angiogenesis studies was not yet satisfactorily tested. Extensive tests are necessary because of adverse factors such as diffusion and convection of the agent in the vessels [14-17].

Nanoparticles consisting of high-Z elements are good candidates as angiography contrast agents [7,18-20], with or without surface modifications. Gold nanoparticles are particularly interesting [21-27] for a number of reasons including: the potentially good biocompatibility, the ample possibilities for surface chemistry manipulations and the recent discovery of new and powerful irradiation-based methods for the fabrication of dense, stable, and mono-dispersed colloids [19,20,28,29].

These factors justified the present study. We obtained very good results *in vivo *with bare-AuNPs in conjunction with heparin, specifically the detection of small (3-5 μm) blood vessels, whereas the tests were less positive for AuNPs coated with mercaptoundecanoid acid (MUA) [30] and for commercial (ExiTron^® ^Nano 6000) colloidal nanoparticles [31].

We selected for our tests MUA since it is a widely used coating agent, for example to control the nanoparticle size. ExiTron nano 6000 is a commercial alkaline earth metal-based contrast agent used for preclinical computed tomography. We decided to study bare-AuNPs both alone and in conjunction with heparin, since this is as an anti-clotting and fluidizing agent, preventing nanoparticle aggregation.

We preferred catheter-supported local injection rather than intravenous injection since the latter is more effective for systemic imaging of large areas, whereas in our case we targeted localized imaging--e.g., a sub-cutaneous tumor--and a large nanoparticle concentration inside local small vessels. As a noteworthy side result, potentially interesting for the study of tumor angiogenesis properties [4,9,32,33], bare-AuNPs with heparin also revealed the diffusion of nanoparticles at leakage locations of the microvessels.

## Results and discussion

The tests were performed with the aforementioned types of AuNP contrast agents: MUA-coated, commercial colloidal and bare, alone or with heparin injection. For each nanoparticle type, we performed two series of tests: *in vivo *microradiological imaging in real time, followed by high resolution X-ray microscopy imaging of fixed specimen (10-30 μm slices) from the same animals. The first tests probed the capability to detect the smallest vessels *in vivo*; the second analyzed in detail the nanoparticle spatial distribution. The imaging procedure included in some cases tomographic reconstruction.

**Figure 1:**
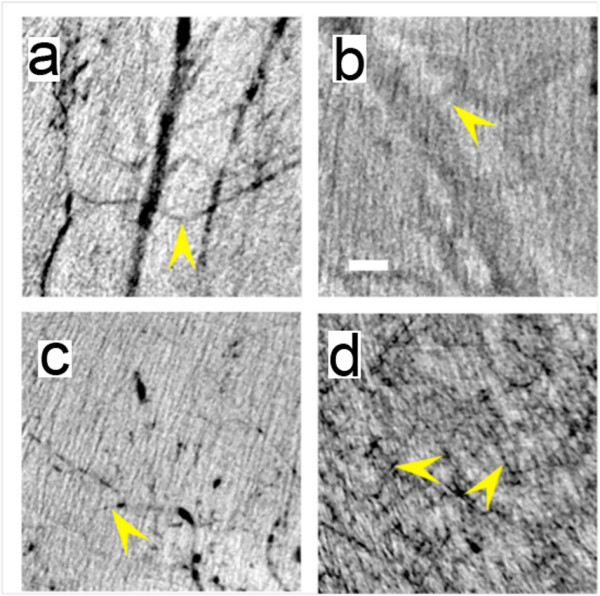
**Direct comparison of the performances of the different tested types of AuNPs in imaging very small vessels**. *in vivo *X-ray micrographs taken in the leg region with (a) MUA-coated AuNPs, (b) commercial ExiTron^® ^Nano 6000, (c) bare-AuNPs and (d) bare-AuNPs with heparin. All images in our study were taken immediately after the corresponding injections. The arrows mark the smallest observable vessels: the measured diameters are 20 μm in (a), 88 in (b), 15 in (c) and 6 in (d). The scale bar of Figure 1b, 200 μm, is valid for all four panels

Figure 1 shows an overall performance comparison of the different types of nanoparticles. The arrows mark some of the small detectable vessels in the *in vivo *images: (a) 20 μm diameter for MUA-coated AuNPs, (b) 88 μm for commercial ExiTron^® ^Nano 6000, (c) 15 μm for bare-AuNPs and (d) 6 μm for bare-AuNPs plus heparin injection. It is qualitatively clear that the ultimate detectable vessel size changes significantly from one particle type to another.

The first part of our study concerned MUA-coated AuNPs with an average size ~4 nm. Potentially, such nanoparticles have good physical characteristics for high-resolution imaging: small but mono-dispersed size and good colloidal stability. The tests, however, were only partially satisfactory.

**Figure 2:**
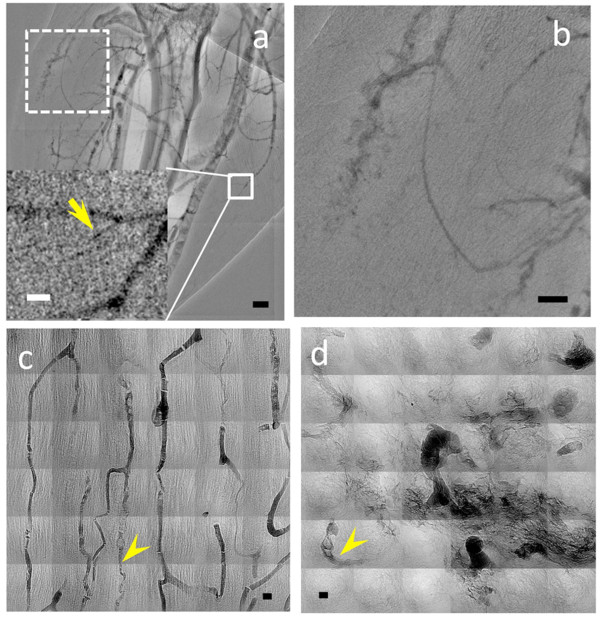
**X-ray micrographs of the microvasculature of the leg area of a tumor bearing mouse taken after injection of MUA-coated AuNP**. *In vivo *snapshots like (a) were taken from a sequence of microradiology images 5 min after the injection; the vessel size marked by the arrow in the inset is ~10 μm. (b) Magnified images of the dotted-line square portion of (a) showing a region of extravascular diffusion of the contrast agent. The scale bars are (a) 500 μm, (b) 250 μm, inset in (a) 50 μm. (c) and (d) are high resolution X-ray microscopy images of MUA-coated AuNPs in muscle and in tumor microvessels. Some microvessels with rather small diameter, ~3 μm, are marked by arrowheads. Substantial extravascular diffusion is found in tumor area. The scale bar in (c) and (d) is 5 μm

On one hand, Figure 2a and 2b show that MUA-coated AuNPs can be imaged *in vivo *and in real time (Additional file 1: Figure S1) to delineate the major microvasculature and microvessels down to < 20 μm in diameter (inset of Figure 2a). We detected no particle aggregation, which could otherwise affect the flow and filling of very small microvessels and the microvasculature perfusion--that is also visible in the tumor part of the tissue. On the other hand, most parts of the microvasculature, including those expected in normal areas, are missing from these images.

We used high-resolution microscopy to check, both for the normal and cancer regions, if the MUA-coated AuNPs remained in the capillaries or diffused out of them. The result, shown in Figure 2c and 2d, was that they remained in the normal capillaries of non-cancer tissues (Figure 2c), but leaked out of the anomalous capillaries of cancer regions (Figure 2d).

It is also qualitatively clear that the lowest vessel diameters in the well-ordered microvasculature of normal tissues (Figure 2c) are substantially smaller than those detectable in Figure 2a and 2b. Likewise, Figure 2d shows very small vessels in a tumor area (marked by the arrowhead), whereas no vessel smaller than tens of μm can be detected in the tumor area in Figure 2a and 2b. This difference can be attributed to the relatively low Au concentration of the MUA-AuNP colloid that does not provide sufficient contrast for *in vivo *imaging (while high resolution X-ray imaging of fixed specimen is not affected by this problem). Whatever the cause, our empirical conclusion is that this type of particles is not suitable for *in vivo *detection of very small capillaries, due to insufficient contrast.

As to the leakage of MUA-AuNP out of the microvessels as seen in Figure 2b and confirmed by Figure 2d, we can argue that the nanoparticles size is large enough to avoid free diffusion, but also small enough to diffuse through the basal membrane apertures observed in abnormal cancer capillaries. Most importantly, these results directly reveal the leaking of nanoparticles out of tumor microvessels, a proposed reason for the differential nanoparticle accumulation at tumor areas.

**Figure 3:**
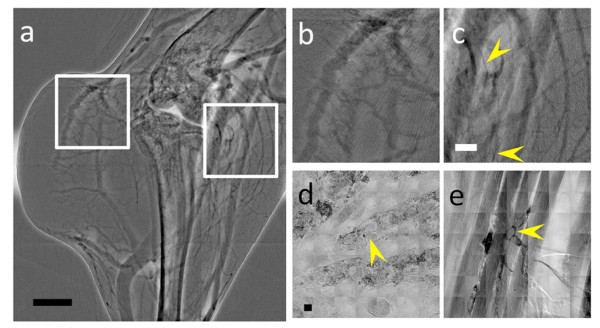
**X-ray micrographs of the microvasculature of the leg area of a tumor bearing mouse, taken with the ExiTron^® ^Nano 6000 contrast agent**. (a) is an *in vivo *microradiology image taken 4 min after the agent injection. (b) and (c) are magnified images of the square regions in (a) corresponding to a tumor area (b) and to a normal tissue area (c). Some small vessels of diameter ~23 μm are marked by the arrowheads. (d) and (e) are high resolution X-ray images showing microvessels in subcutaneous tissue and muscle vessels The arrowheads mark examples of the smallest vessels, ~20 μm in (d) and ~8 μm in (e). Scale bars: 1 mm (a), 500 μm (b and c) and 10 μm (d and e)

The second tested contrast agent was the commercial product ExiTron^® ^Nano 6000 (average nanoparticle size ~110 nm), currently used for small-animal angiography. The results were again rather negative: we could image only vessels with diameter larger than 23 μm (Figure 3a-3c). The high-resolution X-ray microscopy images (Figure 3d and 3e) showed that these nanoparticles were indeed perfused into subcutaneous tumor and muscle vessels. Therefore, the reason why this agent failed to image the smallest vessels was again low contrast. Furthermore, no extracellular diffusion was detected, as expected for such large nanoparticles.

**Figure 4:**
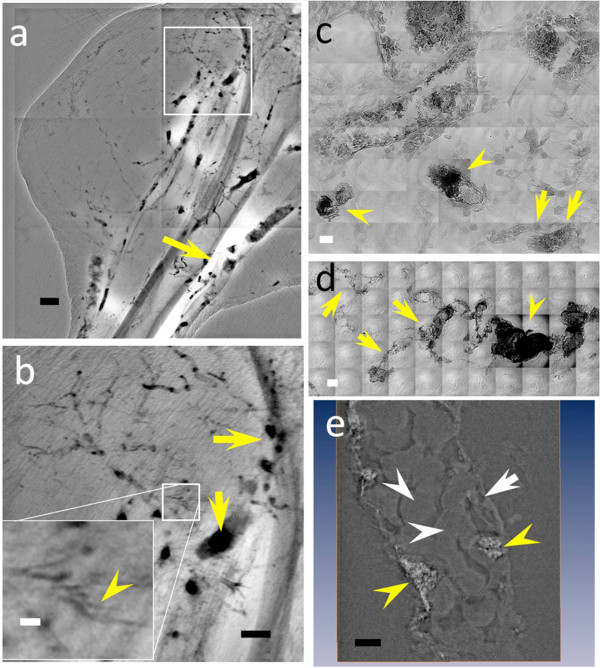
**(a) and (b) are *in vivo *X-ray micrographs of the microvasculature of the leg region of a mouse taken with bare-AuNPs**. The smallest detected microvessel has ~8.6 μm diameter, marked by the yellow arrowhead in the inset of (**b**). Note that the AuNP distribution in the vessels is not continuous. The yellow arrows emphasize that the nanoparticles aggregated in the vessel into large clusters, eventually blocking the flux. (**c**), (**d**) and (**e**) are high resolution X-ray images of vessels in subcutaneous tissue partially coated by bare-AuNPs. Yellow arrowheads indicate agglomerated AuNPs, the white arrow marks an endothelial cell nucleus and the white arrowheads mark erythrocytes in the vessels. Most of the bare-AuNPs adhere to vessel walls and do not interact with erythrocytes. The scale bars are (**a**) 500 μm, (**b**) 250 μm (inset 50 μm) (**c**) and (**d**) 10 μm and (**e**) 2.5 μm

The third series of tests was conducted on bare-AuNPs. These nanoparticles are known to be unstable in blood: they agglomerate and obstruct vessels. Our bare-AuNP colloids were prepared by reduction with intense X-ray irradiation, which provides much better colloidal stability in blood than the citrate reduction method [28]. However, Figure 4a (the sequential images are shown in Additional file 2: Figure S2) and the magnified square area shown in 4b reveal that these AuNPs are not completely immune from problems, particularly in the blood stream inside microvessels, and do agglomerate forming clusters. High-resolution X-ray microscopy images (Figure 4c and 4d) show that the vessels are at least partially coated.

Tomographically reconstructed X-ray images (Figure 4e and Additional file 3: Figure S3, Additional file 4: Video S1, Additional file 5: Video S2) confirm that the bare-AuNPs adhere to the vessel wall while forming clusters, eventually blocking the flux inside the vessels (yellow arrowheads in Figure 4e). This behavior does not allow imaging very small vessels and creates artifacts for *in vivo *microangiography, due to aggregation and obstruction. The detectable vessel size limit for fixed specimens was approximately 8 μm, substantially better than that of ExiTron, and the density of the revealed microvessels was higher than for MUA-AuNP. The high concentration of small (~20 nm) nanoparticles yielded by our fabrication method may explain this better performance, since it allows the nanoparticles to be perfused and to aggregate only in the microvessels.

To prevent the formation of large clusters and make it possible to detect even smaller vessels, we injected bare-AuNPs after perfusion of the anti-coagulant heparin. The results were more positive than those of the other tests. No large clusters were observed and the AuNPs distributed through micro-vessels without obstruction, enabling the detection of < 6 μm capillaries in fixed specimens.

**Figure 5:**
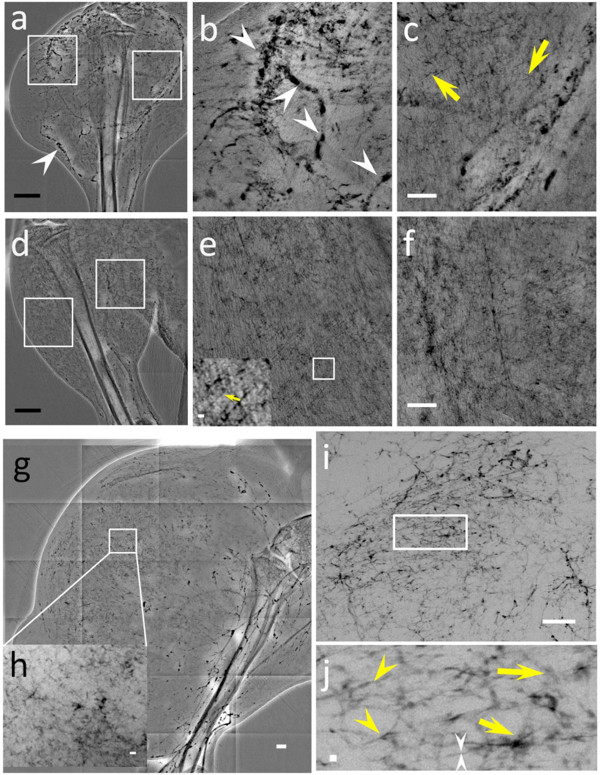
**X-ray micrographs showing the microvasculature of normal tissue and tumors at different time after the tumor inoculation, taken with bare-AuNPs and heparin injection**. (a) *In vivo *image of the lateral thigh, 7 days after inoculation. (b) and (c) magnified images of the left square in (a), near the tumor area, and of the right square, corresponding to normal tissue area (medial thigh). The arrowheads in (a) and (b) mark vessels showing AuNP agglomeration while the yellow arrows mark vessels of < 6 μm diameter. (d) *In vivo *image of the normal lateral thigh. (e) and (f) magnified images the left and right squares in (d). The inset in the lower left corner of (e) is an additionally magnified image of its small square and the yellow arrow marks a < 6 μm diameter vessel. (g) *In vivo *image of the lateral thigh, 16 days after inoculation. (h) magnified image of the square in (g). (i) image of a 1 mm thick tissue removed from the thigh shown in (g). (j) Magnified image of the rectangle in (i); the yellow arrowheads mark abnormal vessels, the white arrowheads a vessel with ~2 μm diameter and the yellow arrows areas with diffusion of bare-AuNPs. Scale bars: (a), (d) and (g): 2 mm; (b), (c), (e), (f), and (i): 500 μm; inset of (e) and (h): 50 μm and (j): 10 μm

Figure 5 shows that the AuNPs were indeed uniformly distributed throughout the vessels of the tumor and normal leg tissues. Some agglomerations appeared in tumor areas (marked by arrowheads in Figure 5b) without, however, preventing the detection of very small vessels--such as the one marked by the yellow arrows in Figure 5c and in the inset of Figure 5e. This limited agglomeration is reasonable since the tortuous character and inhomogeneous diameter of tumor vessels can cause aggregation and accumulation of our dense contrast agent.

We also verified that there was no heparin-induced modification of the vessels morphology that could otherwise interfere with our image analysis. Note that our bare-AuNPs (1.57 mg/ml concentration) are well dispersed in distilled water but immediately precipitate in PBS. The addition of 500 U/ml of heparin in the PBS eliminated the precipitation: the nanoparticles were as well dispersed as in distilled water (Additional file 6: Figure S4). However, we also performed tests with bare-AuNPs pre-mixed with heparin and the results in detecting small vessels were much less satisfactory due to the dilution of the nanoparticle concentration in the pre-mixed solution. Further tests (underway) are needed to check if possible physiological effects of co-injected heparin contributed to the enhanced vessel detection.

The success of bare-AuNPs with heparin illustrates the importance of the trade-off between particle aggregation--which enhances the X-ray contrast--and the uniform distribution required to image all details of the tumor microvasculature. In essence, with heparin the aggregation of bare-AuNPs was found to be limited even in very fine vessels.

Figure 5 also shows that by using bare-AuNPs with heparin we could observe important details of the tumor micro-vasculature. Specifically, more capillaries were detected in muscle tissues (Figure 5c, e and 5f) near the cancer area than in the tumor subcutaneous tissue (Figure 5b). In the late stages of subcutaneous tumor development, we could also see the angiogenesis-related formation of capillaries in the tumor center (Figure 5g). Such results provide useful information about the dynamics of tumor development.

**Figure 6:**
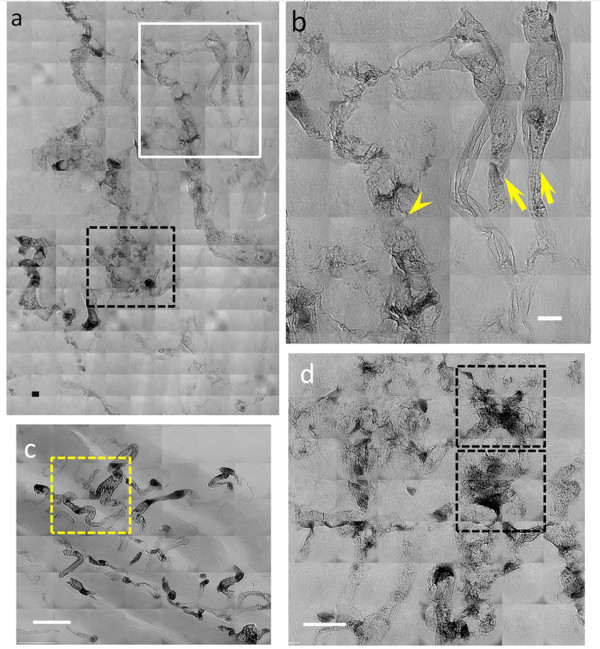
**High resolution X-ray images of a 7 day tumor in a mouse after heparin treatment and bare-AuNP injection**. (**a**), (**b**) and (**d**) are images taken from subcutaneous tumor areas whereas (**c**) refers to a normal tissue region. The two arrows in (**b**) mark the normal vessels; the arrowhead marks tumor vessels that show extravascular diffusion of the bare-AuNPs. (**d**) shows abnormal microvasculature, especially in the two marked squares, with bare-AuNPs diffused out of the microvessles. Scale bars: 10 μm (**a **and **b**) and 25 μm (**c **and **d**)

Tomography images (Additional file 7: Video S3) further confirmed the positive results of the heparin tests. They showed indeed that bare-AuNPs accumulated in the tumor vessels. In high-resolution images, the AuNPs coated the vessel wall and in some cases accumulated in tortuous vessels. With heparin, the distribution of bare-AuNPs delineated the vasculature (Figure 6), especially for abnormal vessel areas.

As shown in Figure 6 and Additional file 8: Figure S5 and Additional file 9: Video S4, we could also detect the diffusion of AuNPs from tumor capillaries. Tumor vessels presented a highly disorganized and tortuous vascular morphology with irregularly varying diameter along their length. Numerous "open windows" (probably due either to endothelial fenestrae or basal membrane disruptions, consecutive to the lack of pericytes, loss of inter-endothelial tight junctions and other possible causes), could be clearly imaged in the vicinity of diffusion areas of bare-AuNPs (the black square area in Figure 6a and 6d). These are symptoms of extravascular diffusion occurring in blood capillaries abnormally formed due to the tumor angiogenic stress. Therefore, images like those of Figure 6 can illustrate this effect in three dimensions and allow the analysis of detailed features. In contrast, normal muscle vessels presented no symptoms suggesting extravascular AuNP diffusion, indicating that the vessel walls were well organized (Figure 6c).

In summary, our tests demonstrate the importance of the nanoparticle preparation procedure when the objective is to image *in vivo *all the details of micro-vasculature, down to the smallest ones. Furthermore, they indicate that the use of X-ray imaging for microvasculatures requires a close attention to chemical properties of contrast agents. On the negative side, we found that MUA-coated AuNPs, ExiTron^® ^Nano 6000 and bare-AuNPs *per se *cannot lead to the detection of the smallest capillaries. On the positive side, this detection was made possible by the co-injection of bare-AuNPs with heparin, preventing nanoparticle aggregation and potential obstruction of small blood vessels. This enabled us, for the first time, to detect by X-ray imaging *in vivo *capillaries with diameter substantially smaller than 10 μm.

We put our conclusions about the smallest detectable vessels *in vivo *on quantitative ground. We used the vessel contrast parameter defined as *C *= *[(I_max_-I_min_)/(I_max_+I_min_)]/R*, where *I*_max_, *I*_min _are the maximum and minimum pixel values for a line crossing the vessel [34] and *R *is the average noise level for the probed area. A vessel is detectable as long as *C *is larger than a threshold value. Empirically, we estimated this threshold to be 0.5-1.5.

For in *vivo *imaging, the quantitative evaluations were extracted from the following statistical sets. 25, 16, 17 and 19 vessels were measured on each mouse for bare-AuNPs with heparin, bare-AuNPs alone, MUA-AuNPs and the Exitron^®^. Three mice were tested for each case.

**Figure 7:**
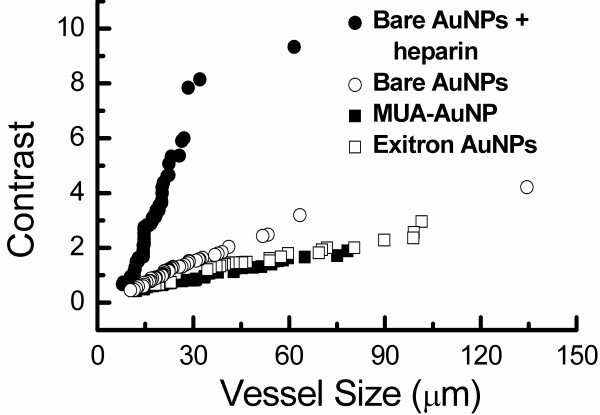
**Plots of the contrast C-parameter (defined in the text) vs the vessel diameter extracted from *in vivo *images for the different investigated cases**. It is clear that bare-AuNPs with heparin lead to the detection of smaller vessels than the other AuNP species

We extracted from the images the average *C*-value corresponding to each measured vessels size; the results are shown in Figure 7. The behavior is generally linear except for bare-AuNP with heparin above ≈30 μm. Linearity can be easily understood assuming, for simplicity, cylindrically shaped vessels. The deviation for bare-AuNPs with heparin can be explained assuming that for large sizes such nanoparticles can flow smoothly through the vessels, with less aggregation on the vessel wall and a weaker contribution to the contrast.

From the plots of Figure 7 we can quantitatively assess the minimum detectable size. Setting the threshold *C*-value at 0.75, the smallest vessels visible with bare-AuNPs plus heparin have a diameter ≈9 μm. With bare-AuNPs the smallest diameter is 15.5 μm; with MUA-AuNPs or ExiTron^®^, it becomes 23 μm. Slightly larger detectability thresholds *C *= 1 and *C *= 1.5 correspond, in the four cases, to minimum vessel diameters of 11.5, 20, 34 and 24.5 μm, and of 12.8, 29, 55 and 50 μm. These results quantitatively confirm our qualitative conclusions.

By best fitting the plots of Figure 7, we found that the slope for bare-AuNPs with heparin is 6.8 times larger than without heparin, and approximately 15 times larger than for MUA-AuNPs and Exitron^®^. The heparin-induced large slope enabled us to detect more vessels in a given area: 83 per mm^2 ^on the average, compared to 27, 28 and 13 for bare-AuNPs alone, MUA-AuNPs and the Exitron AuNP contrast agent.

Similar conclusions can be drawn for the fixed samples: the slope for bare-AuNPs with heparin is 1.9, 2.2 and 5.8 times larger than for bare-AuNPs alone, MUA-AuNPs and Exitron^®^. However, since the contrast agent can be drained out during the fixation process, the contrast-vessel size relation is no longer strictly linear. The detected vessels per mm^2 ^were 2.38 × 10^3 ^for bare-AuNPs with heparin and 0.39 × 10^3^, 1.0 × 10^3 ^and 0.43 × 10^3 ^for the other three cases. One should note that fixed samples are less vulnerable to radiation damage and high contrast images can be obtained with long acquisition times. It is even possible to perform three-dimensional tomography reconstruction to allow very accurate measurements of the vessel size.

The mechanism that makes bare-AuNPs with heparin successful is not entirely clear, so that our result should be considered as experimental evidence, to be further investigated. At this point, we can suggest that the good dispersion properties of MUA-coated AuNPs and ExiTron^® ^nanoparticles allows them to diffuse in the blood without aggregation. The absorption contrast is not sufficient to cause easily detectable delineation of small blood vessels. However, the relatively unstable bare-AuNPs agglomerate and adhere selectively on the walls of small vessels. These stationary high-contrast agglomerates produce much higher contrast than the diluted nanocolloids.

As a corollary result, we found that the co-injection of bare-AuNPs and heparin also allowed the detection of symptoms of the extracellular diffusion due to angiogenesis stress. This is an important result on its own since it opens the way to detailed studies of the rheological properties of tumor blood capillaries as well as the determination of capillary fenestration or morphological aberrations of vessel walls.

We note that comparable progress in pathologic vascular detection was achieved by Fourier transform infrared (FTIR) imaging [35]. Combined with the present progress in X-ray imaging, these two techniques significantly enhance the capability to analyze small blood vessels on a microscopic scale.

## Conclusions

The future applications of the above optimal contrast-enhancement method--bare-AuNPs with heparin--depend to some extent on the feasibility of using synchrotron sources for *in vivo *studies of large animals. There is ample evidence that tests can be conducted on live animals, and we detected no image changes as a consequence of radiation effects (up to 5 s for taking the high-resolution images). Therefore, we can conclude that angiography with our most effective procedure is feasible in principle for large live animals. Some experience also exists on the use of synchrotron techniques for live patients, but any conclusion in that sense would be premature at this stage.

## Methods

### Cell culture

EMT-6 cells were obtained from American Type Culture Collection (ATCC) and cultured at 37°C in humidity air atmosphere with 5% CO_2_. EMT-6 cells were incubated with Dulbecco's Modified Eagle's Medium: Nutrient Mixture F-12 (DMEM/F12)/10% fetal calf serum (FCS) was used as growth medium. All mediums were purchased from GIBCO^®^.

### Tumor development

1 × 10^7 ^EMT cells/ml in phosphate buffer saline (PBS) 50 μl were inoculated in the subcutaneous tissue of left leg region for 7 days to develop subcutaneous tumors. All the procedures involving animals were approved by the Academia Sinica Institutional Animal Care and Utilization Committee (AS IACUC), Approval Number: Protocol #RMiPHYHY2010039. BALB/cByJNarl mice were provided by National Laboratory Animal Center, Taiwan. All mice were housed in individual ventilated cages (five per cage) with wood chip bedding and kept at 24 ± 2°C with a humidity of 40%-70% and a 12-hour light/dark cycle. The subcutaneous tumor volume was estimated as v = 0.5 × a × b^2^, where a and b are the smallest and the largest diameters. Tumor imaging was started after about 7 days, when the tumors reached a volume of 100 to 120 mm^3^.

### Contrast agents

For the different tests we used ~200 μl of different contrast agents: colloidal solutions of bare-AuNPs, MUA-modified AuNPs and ExiTron^® ^Nano 6000 nanoparticles. All colloids except ExiTron were synthesized with the X-ray irradiation method described in Refs[12,18]. ExiTron^® ^nano 6000 (Viscover^®^), was purchased from Miltenyi Biotech GmbH (Berlin, Germany). The colloidal concentrations and average mean hydrodynamic diameter were: 15.76 mg/ml and 15.5 ± 5.1 nm for bare-AuNPs, 31.52 mg/ml and 3.91 nm MUA-modified AuNPs, and 160 mg/ml and 110 nm for ExiTron^® ^Nano 6000 nanoparticles.

We analyzed the nanoparticle concentration by inductively coupled plasma optical emission spectrometry (ICP-OES, Perkin Elmer Optima 2000 DV, Norwalk, CT). A particle size analyzer (90 plus, Brookhaven Instruments Corp., Long Island, USA) determined the hydrodynamic diameter of the nanoparticles. We performed TEM measurements in a JEOL JEM-2100F system with a 4,096 × 4,096 CCD imaging system (Gatan, UltraScan 4000) operated with an accelerating voltage of 200 kV. The specimens were prepared by placing a drop of solution on a carbon-coated copper grid and dried at 40°C. We also confirmed the nanoparticle size by TEM measurements, showing that the shapes were all spherical.

PE-08 catheters (BB31695, Scientific Commodities, Inc., I.D.: 0.2 mm, O.D.: 0.36 mm) were used to inject the contrast agents. The catheter was placed under anesthesia induced by intramuscular injection of 10 μl of Zoletil 50 (50 mg/kg; Virbac Laboratories, Carros, France) per mouse (weight ~20-25 g). For heparin treatment, 20 μl of heparin 5000 U/ml were injected via the tail vein before contrast agent injection. The anterior tight skin was incised along a 1-cm^2 ^circle and after a sharp dissection; the catheter was inserted into the femoral artery and secured by a 6-0 nylon ligature. With the mouse in the imaging position, one of the contrast agents was injected at a 1 μl/s rate. During imaging the mice were kept under anesthesia using 1% isoflurene in oxygen.

### Tissue slice preparation

Seven days after the tumor cell inoculation and after sacrifice, the tumors were removed from the subcutaneous tissue and the lungs. The specimens were immersed in 3.7% paraformaldehyde for 24 hr. After fixation, the specimens were embedded in paraffin and sliced. The specimens were stained by heavy metal staining for X-ray imaging. The stained specimens were washed by distilled water 3 times for 5 minutes, dehydrated by washing with increasing ethanol concentrations, embedded in Embed-812 Resin (EMS, Hatfield, PA).

### X-ray imaging

Micro-radiology was first implemented with unmonochromatized (white) synchrotron X-rays emitted by the 01A beamline wavelength shifter of the National Synchrotron Radiation Research Center (Taiwan) [36]. The photon energy ranged from 4 keV to 30 keV with a peak intensity at energy ~12 keV and the beam current was kept constant at 300 mA with the top-up operation mode. To obtain 3 × 3 mm images, the X-rays were first converted to visible light by a CdWO_4 _single crystal scintillator and then captured by an optical microscope with a CCD camera (model 211, Diagnostic instruments, 1,600 × 1,200 pixels). To limit the risk of damage, the radiation dose was reduced by > 100 times by attenuating the emitted X-ray beam with a 1.1 mm of silicon. The dose was 33.9 Gy per 100 ms for a specimen thickness of 1 cm placed before the animal.

The exposure time was ~100 ms and the distance between the sample and the scintillator was ~15 cm; a 2× lens in the optical microscope was used to obtain the desired field of view. The size of each pixel in the final image taken with the 2× lens was ~2.8 × 2.8 μm^2^. A simple background flattening image filter was used for large area micro-radiology images such as those of Figures 2(a) and 3(b), 3(a)-(c), 4(a), (b) and 5(a)-(j).

High-resolution images were taken on the 32-ID transmission X-ray microscopy (TXM) beamline of the Advanced Photon Source (APS) at the Argonne National Laboratory. The full-field TXM uses a set of capillary condensers that provide fitting illumination of the object, having a numerical aperture matched to a set of zone plate lens objectives. The condensers are elliptically shaped glass capillaries. The inner diameter of 0.9 mm was chosen to maximize the vertical acceptance of the APS undulator beam at 65 m from the source. The estimated monochromatic X-ray flux focused by the condenser (after a Si (111) double crystal monochromator) was 2 × 10^11^/s at 8 keV.

The high brightness of the APS and the optimized condensers design yielded an excellent imaging throughput of 50 ms/frame with ~1 × 10^4 ^CCD counts per pixel. The microscope system could also operate in the Zernike phase contrast imaging mode with a gold Zernike phase ring placed at the back focal plane of the zone plate objective. This mode increases the contrast for fine features in the hard X-ray spectral region [37-40].

## Competing interests

The authors declare that they have no competing interests.

## Authors' contributions

CCC; HHC; YSC conducted the experimental work and part of the data analysis; SFL; KCW; XC prepared the nanoparticle contrast agents and their administration in animal; YH; CP; GM analyzed the data and wrote the final version of the manuscript. All authors read and approved the final manuscript.

## Supplementary Material

Additional file 1 **Figure S1**. Sequential images of MUA-coated AuNPs injected in the femur artery. Only large vasculature can be imaged. Top: sequence of microradiology images of a mouse leg at different times after injection of 200 μL 31.52 mg/ml of 2.18 ± 0.51 nm MUA-coated AuNPs. Small vessels are not fully visible. Pictures (a)-(e) were taken at 1 min intervals starting 60 s after injection: Middle: the same images as in the top row, after image processing with a background flattening filter. The vessel in the magnified portion in (j) is ~10 μm. Bottom: magnified images of the square area in (j). Scale bars: (a)-(j) 500 μm; (k)-(o) 250 μm; (j) (magnified portion) 50 μm. Click here for file

Additional file 2 **Figure S2**. Sequential images of bare-AuNPs injected in the femur artery: agglomerations were clearly observed (Figure 3). The images were taken after injection of 200 μL 15.76 mg/ml of 15.5 ± 5.1 nm bare-AuNPs in the leg. The interval between images is ~60 s. The images (f)-(o) were processed with a background flattening filter. The vessel in the magnified portion in (o) is ~8.6 μm (yellow arrowhead). Yellow arrows indicate that the nanoparticles adhere to the vessel wall while forming clusters, eventually leading to the complete blockage of the flux inside the vessels. Scale bars: (a)-(e) 500 μm, (k)-(o) 250 μm, (o) (magnified portion) 50 μm. Click here for file

Additional file 3 **Figure S3**. High resolution images showing bare-AuNPs accumulated in small vessels without heparin treatment. (a) and (d) are projection images; (b) and (g) tomographically reconstructed pictures; (c), (e), (f) and (i) are slices of reconstructed images. (h) is a combination of reconstructed slice images. Bare-AuNPs accumulated in small vessels can be seen in (a), (d) and (g). Most of the bare-AuNPs did agglomerated--see (c), (e) and (i) (the white arrows points to agglomerated nanoparticles)--and adhered to the vessel walls as seen in (c) and (f) (the yellow arrowheads point to the nucleus of endothelial cells). There are no interactions with erythrocytes (marked by white arrowheads in (c), (e), (f) and (i)). Bare-AuNPs are also seen on the surface of white blood cells (marked by yellow arrows in (h) and (i)). Scale bars: 2.5 μm. Click here for file

Additional file 4 **Video S1**. Multi projection images of high-resolution X-ray images of bare-AuNPs accumulated in vessels. Click here for file

Additional file 5 **Video S2**. Reconstructed high-resolution images of bare-AuNPs accumulated in vessels. Click here for file

Additional file 6 **Figure S4**. Tests on bare-AuNPs in different solution. Bare-AuNPs in PBS (1.576 mg/ml) precipitate in a short time (left). When bare-AuNPs are added to PBS combined with heparin (1.576 mg/ml in PBS with 500 U/ml heparin), the precipitation does not occur (middle). For comparison, bare-AuNPs suspend well in distilled water (right, 1.576 mg/ml). Click here for file

Additional file 7 **Video S3**. Tomographically reconstructed video of bare-AuNPs with heparin treatment. The movie shows well-distributed bare-AuNPs in vessels (Figure 4i). Click here for file

Additional file 8 **Figure S5**. High-resolution projection images (left) and tomographically reconstructed images (middle and right) of bare-AuNPs with heparin treatment, taken in the cancer area (a)-(c) or in the subcutaneous area (d)-(f). The images (a), (b) and (c) show the leaking of AuNPs outside the cancer vessel area, whereas those in (d), (e) and (f) show them aggregated on the vessel walls. Scale bar: 2.5 μm. Click here for file

Additional file 9 **Video S4**. High resolution X-ray projection images of bare-AuNPs leaking in tumor vessels after heparin treatment (Figure 5d). Click here for file
