# Supplementary figures and images for: Gold nanoparticles as high-resolution X-ray imaging contrast agents for the analysis of tumor-related micro-vasculature
Source: J Nanobiotechnology. 2012 Mar 12;10:10. doi: 10.1186/1477-3155-10-10 (PMC3316138; doi:10.1186/1477-3155-10-10)

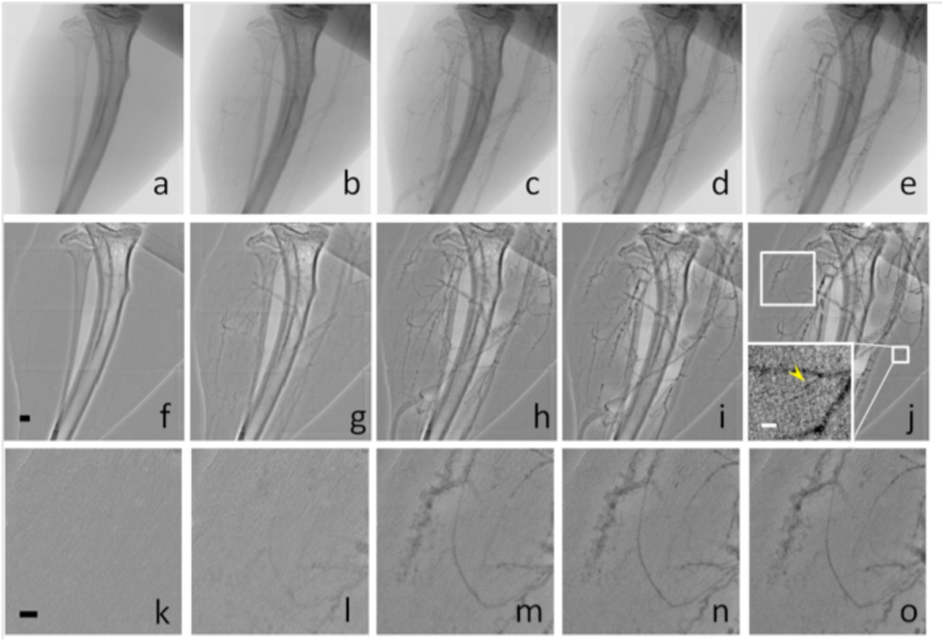

Supplement: Additional file 1 — Figure S1. Sequential images of MUA-coated AuNPs injected in the femur artery. Only large vasculature can be imaged. Top: sequence of microradiology images of a mouse leg at different times after injection of 200 μL 31.52 mg/ml of 2.18 ± 0.51 nm MUA-coated AuNPs. Small vessels are not fully visible. Pictures (a)-(e) were taken at 1 min intervals starting 60 s after injection: Middle: the same images as in the top row, after image processing with a background flattening filter. The vessel in the magnified portion in (j) is ~10 μm. Bottom: magnified images of the square area in (j). Scale bars: (a)-(j) 500 μm; (k)-(o) 250 μm; (j) (magnified portion) 50 μm. [file 1477-3155-10-10-S1.TIFF]

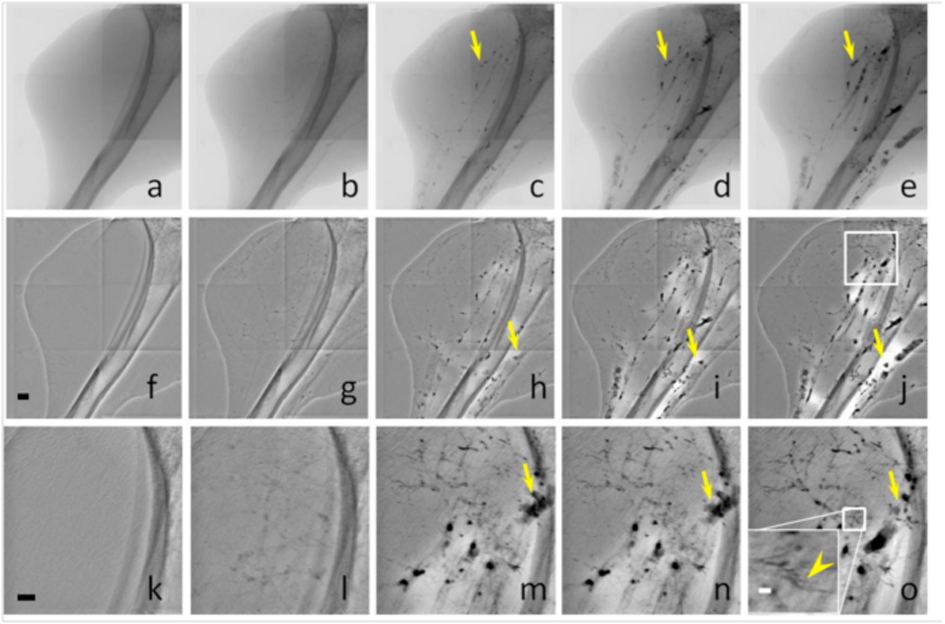

Supplement: Additional file 2 — Figure S2. Sequential images of bare-AuNPs injected in the femur artery: agglomerations were clearly observed (Figure 3). The images were taken after injection of 200 μL 15.76 mg/ml of 15.5 ± 5.1 nm bare-AuNPs in the leg. The interval between images is ~60 s. The images (f)-(o) were processed with a background flattening filter. The vessel in the magnified portion in (o) is ~8.6 μm (yellow arrowhead). Yellow arrows indicate that the nanoparticles adhere to the vessel wall while forming clusters, eventually leading to the complete blockage of the flux inside the vessels. Scale bars: (a)-(e) 500 μm, (k)-(o) 250 μm, (o) (magnified portion) 50 μm. [file 1477-3155-10-10-S2.TIFF]

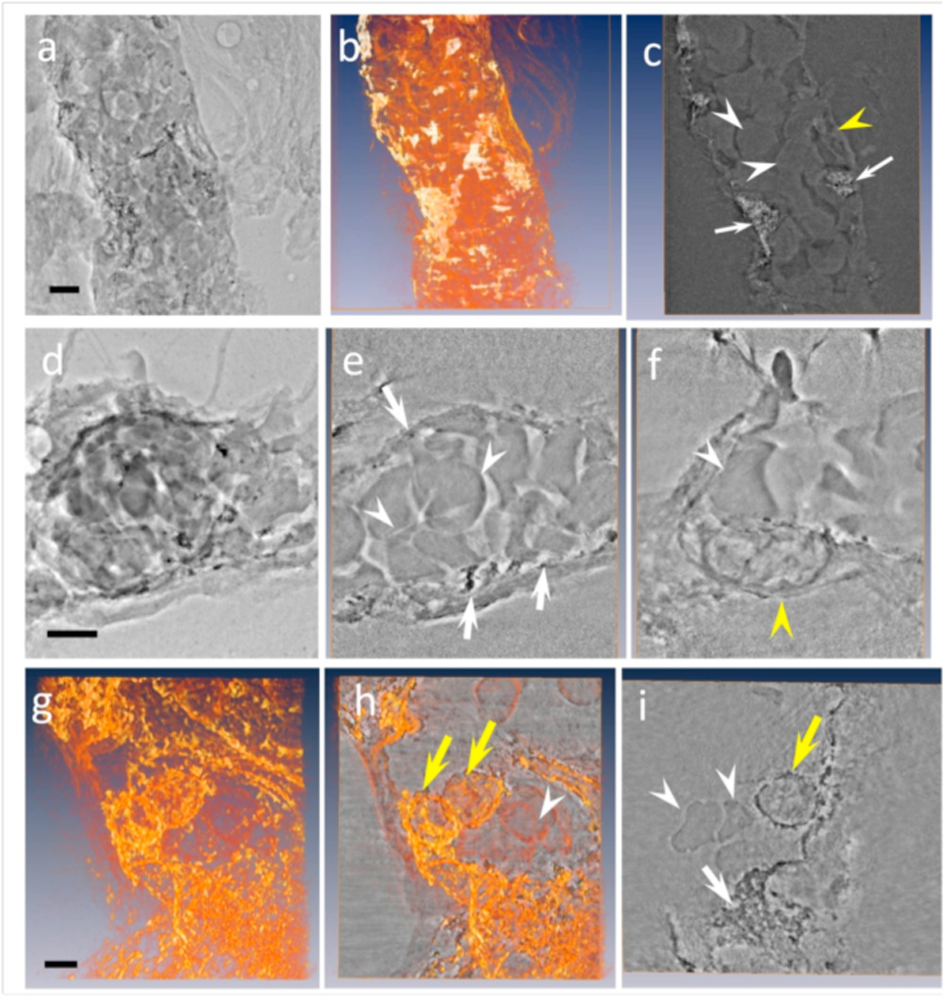

Supplement: Additional file 3 — Figure S3. High resolution images showing bare-AuNPs accumulated in small vessels without heparin treatment. (a) and (d) are projection images; (b) and (g) tomographically reconstructed pictures; (c), (e), (f) and (i) are slices of reconstructed images. (h) is a combination of reconstructed slice images. Bare-AuNPs accumulated in small vessels can be seen in (a), (d) and (g). Most of the bare-AuNPs did agglomerated--see (c), (e) and (i) (the white arrows points to agglomerated nanoparticles)--and adhered to the vessel walls as seen in (c) and (f) (the yellow arrowheads point to the nucleus of endothelial cells). There are no interactions with erythrocytes (marked by white arrowheads in (c), (e), (f) and (i)). Bare-AuNPs are also seen on the surface of white blood cells (marked by yellow arrows in (h) and (i)). Scale bars: 2.5 μm. [file 1477-3155-10-10-S3.TIFF]

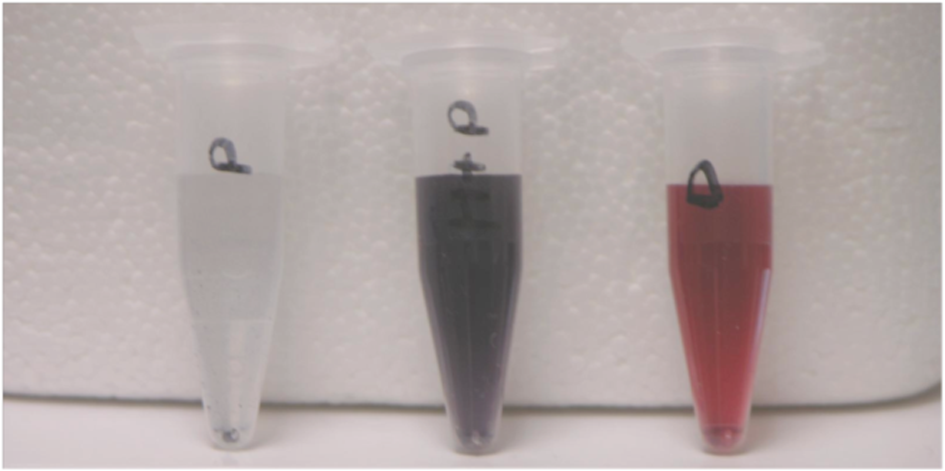

Supplement: Additional file 4 — Video S1. Multi projection images of high-resolution X-ray images of bare-AuNPs accumulated in vessels. [file 1477-3155-10-10-S4.TIFF]

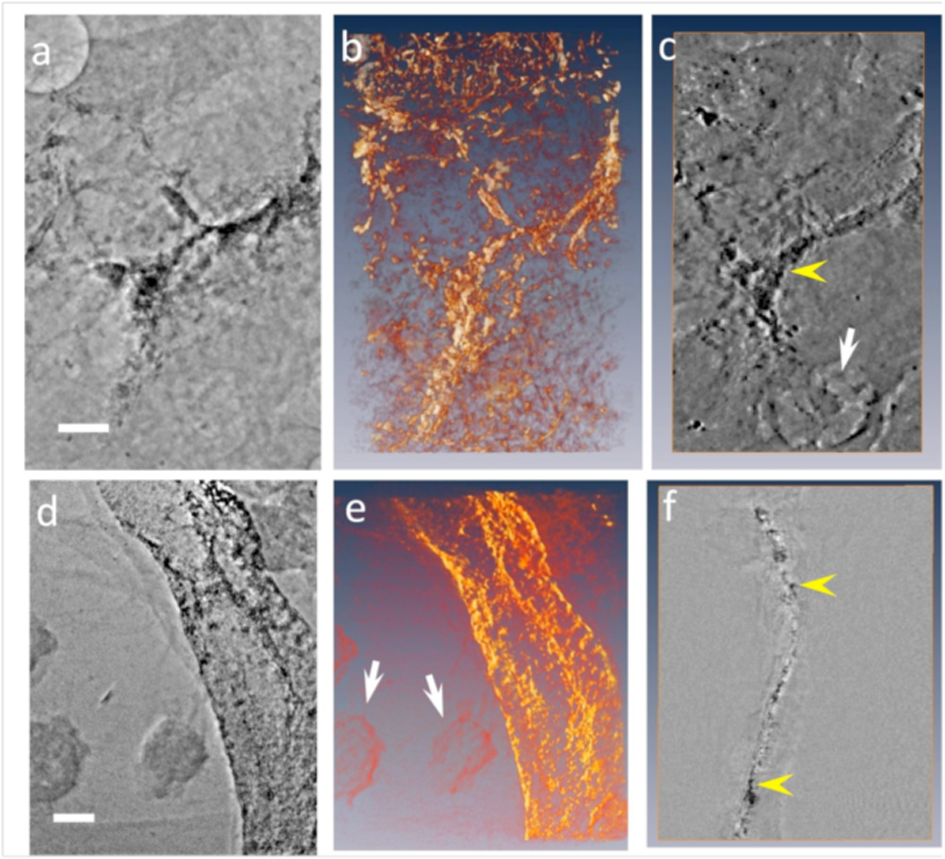

Supplement: Additional file 5 — Video S2. Reconstructed high-resolution images of bare-AuNPs accumulated in vessels. [file 1477-3155-10-10-S5.TIFF]
